# Norplant^™^ Cellulitis

**DOI:** 10.1155/S1064744996000622

**Published:** 1996

**Authors:** David E. Soper

**Affiliations:** Medical University of South Carolina 171 Ashley Avenue Charleston South Carolina 29401-2233 USA


					Infectious Diseases in Obstetrics and Gynecology 4:312 (I 996)
(C) 1997 Wiley-Liss, Inc.
Images in Infectious Diseases in Obstetrics and Gynecology
Section Editor: David E. Soper, MD
NorplantV Cellulitis
David E. Soper, MD
Medical University ofSouth Carolina
Charleston, South Carolina
A 23-year-old woman presented to her gynecologist's of-
rice with a complaint of redness and tenderness at the
site of her Norplan(TM, inserted 2 years prior. One week
prior to presentation she had lacerated her fingertip
while diving in the Bahamas (Fig. 1). Examination re-
vealed erythema and tenderness overlying the Nor-
plantTM site (Fig. 2). A course of orally administered tri-
methoprim/sulfamethoxazole (160 mg/800 rag) twice
daily resulted in resolution of symptoms and signs of
infection. The NorplantTM did not have to be removed.
(C) 1997 Wiley-Liss, Inc.
Images in Infectious Diseases in Obstetrics and Gynecology presents clinically important visual images that a practitioner in women's health
might encounter. If you have a high-quality color or black-and-white photograph representing such an image that you would like
considered for publication, send it with a descriptive legend to David E. Soper, MD, Medical University of South Carolina, 171 Ashley
Avenue, Charleston, SC 29401-2233.
Images is made possible through an educational grant from Pfizer, Inc.
Images in Infectious Diseases in Obstetrics and Gynecology

				

